# The challenges of success: adolescents with perinatal HIV infection

**DOI:** 10.7448/IAS.16.1.18650

**Published:** 2013-06-18

**Authors:** Lynne M Mofenson, Mark F Cotton

**Affiliations:** 1 Maternal and Pediatric Infectious Disease Branch, *Eunice Kennedy Shriver* National Institute of Child Health and Human Development, National Institutes of Health, Rockville, MD 20852, USA; 2 Division of Infectious Diseases, Tygerberg Children's Hospital, Stellenbosch University, Cape Town, South Africa

**Keywords:** perinatal HIV infection, adolescents, HIV care

## Abstract

The great success in the prevention and treatment of pediatric HIV in high resource countries, and now in low resource countries, has changed the face of the HIV epidemic in children from one of near certain mortality to that of a chronic disease. However, these successes pose new challenges as perinatally HIV-infected youth survive into adulthood. Increased survival of HIV-infected children is associated with challenges in maintaining adherence to what is likely life-long therapy, and in selecting successive antiretroviral drug regimens, given the limited availability of pediatric formulations, limitations in pharmacokinetic and safety data of drugs in children, and the development of extensive drug resistance in multi-drug-experienced children. Pediatric HIV care must now focus on morbidity related to long-term HIV infection and its treatment. Survival into adulthood of perinatally HIV-infected youth in high resource countries provides important lessons about how the epidemic will change with increasing access to antiretroviral therapy for children in low resource countries. This series of papers will focus on issues related to management of perinatally infected youth and young adults.

The remarkable success in the prevention and treatment of paediatric HIV infection in high-resource countries has changed the face of the HIV epidemic in children from a fatal disease to that of a chronic illness. With widespread access to antiretroviral therapy in high-resource settings, many perinatally infected children are surviving into adolescence, young adulthood and beyond [1]. With increasing access to antiretroviral therapy in resource-limited settings, a similar population of perinatally infected youth is emerging [2]. Important lessons gained in high-resource settings about how the epidemic changes with increasing access to antiretroviral therapy for children will help to inform management in resource-limited settings [3].

These successes pose new management challenges as perinatally infected youth survive into adulthood. There have been significant difficulties in maintaining adherence to life-long therapy, and in selecting successive antiretroviral drug regimens, given the limited availability of paediatric formulations, pharmacokinetic, and safety data in children and development of extensive drug resistance in multi-drug-experienced children. Long-term survival of youth with perinatal HIV infection has been accompanied by unanticipated needs. These include management of long-term complications of therapy, sexual and reproductive health, mental health needs, and issues of higher education and career training [4,5].

How to transition from complete dependence on adult caregivers and health services provided in paediatric HIV care settings, which are often multidisciplinary, family-centred and include extensive support services, to adult HIV care systems and assuming responsibility for their own care has received little attention [6]. These young adolescents may fall through the cracks and suffer from a sense of abandonment as they lose the familiar and dependable environment and staff of the paediatric HIV clinic (clinicians, social workers, nursing staff) and its support services.

To optimize the psychosocial well-being and treatment outcomes of perinatally infected adolescents and young adults as well as enabling them to lead long, meaningful and productive lives, there is an urgent need to understand the factors that either facilitate or serve as a barrier to the health and well-being of HIV-infected children and youth, and the complications of HIV and therapy as they age. The articles in this series provide a comprehensive evaluation of the issues related to perinatal HIV infection in both high- and low-resource settings.

One common theme is the paucity of research in the area of adolescent perinatal HIV infection, including a lack of epidemiologic data to better define this population of youth on a global basis, and the general lack of data regarding potential impacts of gender on HIV disease in this population.

Sohn and Hazra discuss the changes in the global paediatric HIV epidemic as children receiving antiretroviral therapy age into adolescence, highlighting our lack of knowledge regarding the global numbers of perinatally infected youth over 15 years of age because global reporting does not differentiate between perinatal and behaviourally infected youth [7]. They discuss treatment challenges in multi-drug-experienced children and available data on regional outcomes of treatment and long-term complications in perinatally infected youth in Africa, Asia, the US, Europe and Latin America/Caribbean. They note that the lack of a global surveillance system or mechanism for tracking perinatally infected children as they transition to adulthood results in a lack of understanding of the needs of these children and whether they are retained in care or lost to follow-up.

Agwu and Fairlie discuss the multiple challenges of antiretroviral therapy in perinatally infected youth, with a focus on adherence issues and a review of clinical, immune and viral outcomes [8]. Children with perinatal HIV infection initiate therapy during a period of rapid growth and face decades, if not a life-time, of antiretroviral drug exposure. Perinatally infected youth often have complex clinical histories, multi-class drug experience and often drug resistant virus, complicating their care and limiting choice of therapy. While treatment is life-saving, adherence to therapy is particularly problematic in infected adolescents, with multifaceted aetiologies and little specific research, particularly in resource-limited settings [9]. The authors note that successful treatment in perinatally infected youth is complicated by developmental, cognitive and psychosocial challenges, and that continued successful clinical outcomes of treatment in these youth may be particularly compromised by a resistant virus, non-adherence and the limited pipeline of new agents. Longitudinal data are needed to determine if the increased life-expectancy in treated HIV-infected adults will be duplicated in perinatally infected youth as they transition in to adulthood.

Mellins and Malee discuss mental health issues in perinatally infected youth, reviewing the literature in this area, risk as well as protective factors, treatment modalities and need for further research [10]. Infected youth have a high rate of psychiatric symptoms, particularly attention-deficit disorder and depression, compared to children from similar socio-economic circumstances [11,12]. The aetiology of these disorders is likely multifactorial, including biologic factors such as HIV itself, its treatment, as well as psychosocial factors including chronic illness, poverty, loss of parents, and stigma and rejection by peers. Psychiatric symptoms may be associated with poor behavioural outcomes, such as risky sexual behaviours which could promote HIV transmission (and pregnancy), drug use and poor adherence to therapy. The authors note the critical need for data from and tools for resource-limited settings, and the potential utility of resilience models to identify key areas that may be amenable to preventive interventions.

Laughton and colleagues discuss neurodevelopmental issues in perinatally infected youth, noting that infected youth exhibit problems on general cognitive tests, processing and visual-spatial tasks and are at high risk for psychiatric and mental health problems as discussed by Mellins [10,13]. While antiretroviral therapy has significantly decreased HIV encephalopathy, as HIV-infected children survive into adolescence and young adulthood, more subtle manifestations of central nervous system disease are still seen. These cognitive deficits, problems with attention and psychiatric disorders are far less acutely devastating than encephalopathy but may well have a tremendous impact on these youth as they survive into adulthood. The etiologic factors are complex and may include the effects of HIV infection (both on-going central nervous system viral replication as well as past impact of infection on the developing brain), chronic inflammation, antiretroviral drugs toxic effects, social factors and other exposures (both *in utero* and behaviourally based, such as substance use). The authors note the paucity of data in infected adolescents and in youth from resource-limited settings.

Lipshultz and colleagues discuss the cardiac effects of HIV and its treatment in perinatally infected children and adolescents, including the range of cardiovascular disease in children, the clinical manifestations, pathogenesis and monitoring, including cardiac biomarkers, and treatment [14]. They emphasize the need for routine systematic cardiac evaluation for perinatally infected youth and note that many of the metabolic complications of therapy may also be associated with future cardiovascular disease as the youth age into adulthood.

Barlow-Mosha and colleagues discuss the myriad of metabolic complications of HIV and its treatment that are being observed in youth [15]. While potent antiretroviral therapy has reduced morbidity and mortality, as in adults, long-term metabolic complications are common in infected children. Perinatally infected youth will have prolonged exposure to therapy throughout various stages of growth and development, receive multiple drug regimens as they age, and are at high risk for metabolic complications. High rates of obesity, dyslipidemia and insulin resistance have been described in perinatally infected youth, all of which are factors associated with cardiovascular disease in non-HIV-infected populations [16]. While these youth are still too young to have experienced cardiovascular outcomes, perinatally infected children and adolescents will be subject to the effects of these risk factors as they enter the third and fourth decades of life. The authors note that many of these metabolic toxicities may be asymptomatic and progress unnoticed, particularly in resource-limited settings where monitoring may be limited, and that developing effective strategies to monitor, prevent and manage metabolic complications of therapy in perinatally infected youth will be important, particularly in resource-limited settings.

Puthanakit and Siberry discuss issues related to the effect of HIV infection and antiretroviral therapy on bone. They discuss normal bone development and non-HIV factors affecting development as well as HIV and treatment-related factors impacting bone, and approaches to detection, prevention and management of these problems in youth [17]. Bone undergoes profound changes in size, mass and strength from foetal life to adulthood, and children may be particularly vulnerable to HIV and antiretroviral-related effects on bone due to higher bone turnover; approximately 80% of peak bone mass is attained by age 18–20 [18,19]. Numerous studies have reported lower bone mass in perinatally infected children compared to healthy children of similar age and sex, but the causes, interaction with treatment and risk for fracture are poorly understood [4]. Clinical manifestations of these effects on bone may only become evident as these youth become adults.

Bhimma and colleagues discuss the problem of kidney disease in youth with perinatal infection [20]. They note that while treatment has dramatically reduced HIV-associated nephropathy, other forms of renal disease due to HIV or its treatment have remained. They discuss the spectrum of renal disease that has been reported in children, including renal toxicity from antiretroviral drugs, including pathogenesis, clinical presentation and management.

A number of studies have found that many perinatally infected adolescents may not be aware of their HIV status in both high- and low-resource settings [21–23]. We refer readers to a study by Meless and colleagues, who assessed disclosure among perinatally infected adults in Abidjan, Côte d'Ivoîre [24]. They found a low disclosure rate, particularly for younger adolescents, with only 33% of youth having been informed of their HIV status, and note the need for the development of practical interventions to support age-appropriate HIV status disclosure to children and adolescents.

Weber and colleagues note the emerging data and high prevalence of chronic lung disease in adolescents with perinatal HIV infection, as well as the need for more detailed prospective studies. They note that bronchiectasis and bronchiolitis obliterans are important problems in these children, with lung function tests showing significant impairment. The importance of co-infection with tuberculosis and the emergence of chronic lung disease is discussed, emphasizing the need for early antiretroviral therapy in children to minimize the risk of chronic lung problems. Lastly, they provide guidance for the evaluation of lung health and the need for more prospective data.

This series of articles serves to focus attention on the growing population of perinatally infected adolescents and young adults globally, the complexities of their care, and helps to identify the future research needs. A holistic approach to improve the long-term health of these youth is needed to ensure that our success in achieving survival of HIV-infected children from infancy is maintained into adulthood.
